# Supplementary material for: Targeted proteomics using stable isotope labeled protein fragments enables precise and robust determination of total apolipoprotein(a) in human plasma
Source: PLoS One. 2023 Feb 15;18(2):e0281772. doi: 10.1371/journal.pone.0281772 (PMC9931122; doi:10.1371/journal.pone.0281772)
Supplement: S1 Data — (PDF) [file pone.0281772.s006.pdf]

Reverse Standard Curves

Andreas Hober

2021-06-24

Read Input Data

The input data is read from the data subfolder and gathered into one tibble, `input`.

```
input <- read_csv(params$skyline) %>%
  left_join(read_csv(params$sample_data)) %>%
  left_join(read_csv(params$pool_data))
```

Processing of Data

All columns with data relevant for the plotting of the dilution curves and the further calculations of the linear regressions are collected in one single tibble, `standard_curves`. Additionally, the mean *Ratio To Standard* is calculated for all transitions together with their respective standard deviation and coefficient of variation.

```
standard_curves <- input %>%
  select(prot = `Protein`,
         pep = `Peptide`,
         pool_conc = `Pool Concentration`,
         rep = `Curve`,
         dil = `Dilution Factor`,
         ratio = `Ratio To Standard`) %>%
  mutate(conc = pool_conc/dil) %>%
  mutate(ratio = gsub("#N/A", NA, ratio)) %>%
  mutate(ratio = as.numeric(ratio)) %>%
  filter(!is.nan(ratio) & !is.na(ratio)) %>%
  group_by(prot, pep, dil) %>%
  mutate(mean_ratio = mean(ratio)) %>%
  mutate(std_dev = sd(ratio)) %>%
  mutate(cv = raster::cv(ratio)) %>%
  mutate(fail_cv = cv >= 25 || is.na(cv) || cv == 0) %>%
  arrange(desc(conc))
```

Using the data collected in `standard_curves`, a linear regression and a plot, illustrating the standard curve and its regression, is created for each peptide individually.

```
peptides <- standard_curves %>%
  group_by(prot, pep) %>%
  select(prot, pep) %>%
  distinct()

  curve_tables <- list()
  curve_plots <- list()
  value_list <- list()
```

```
for(i in 1:nrow(peptides)){
  # Reset the linear regression
  lm_temp <- lm(1~1)

  # Create temporary tibble containing data for the evaluated peptide
  temp <- standard_curves %>%
    group_by(prot, pep, conc) %>%
    select(prot, pep, conc, mean_ratio, std_dev, cv, fail_cv) %>%
    distinct() %>%
    filter(prot == peptides$prot[i], pep == peptides$pep[i])

  # Create a filtered tibble with values used for the linear regression
  temp_ref <- cbind(temp[!temp$fail_cv,],
                    pred = rep(NA, sum(!temp$fail_cv)),
                    pred_error = rep(NA, sum(!temp$fail_cv)))

  # Determine the linear regression
  if(nrow(temp_ref) > 2){
    lm_temp <- lm(log2(temp_ref$conc)~log2(temp_ref$mean_ratio))
    temp_ref$pred <- 2^lm_temp$fitted.values
    temp_ref$pred_error <- abs(temp_ref$conc-temp_ref$pred)/abs(temp_ref$conc)
  }

  # refine the linear regression by excluding data points outside the linear range
  while(max(temp_ref$pred_error) > 0.25 && nrow(temp_ref) > 2){
    last_point <- temp_ref$pred_error[nrow(temp_ref)] > temp_ref$pred_error[1]
    if(last_point){
      temp_ref <- temp_ref[-nrow(temp_ref),]
    }else{
      temp_ref <- temp_ref[-1,]
    }
    lm_temp <- lm(log2(temp_ref$conc)~log2(temp_ref$mean_ratio))
    temp_ref$pred <- 2^lm_temp$fitted.values
    temp_ref$pred_error <- abs(temp_ref$conc-temp_ref$pred)/abs(temp_ref$conc)
  }

  # Summarize all data for the peptide into one tibble
  temp <- left_join(temp, temp_ref) %>%
    mutate(linear = !is.na(pred))

  # Store the standard curve tables in a list
  curve_tables[[i]] <- temp %>%
    select(Protein = prot,
           Peptide = pep,
           `Conc.` = conc,
           `Ratio To Standard` = mean_ratio,
           `Standard deviation` = std_dev,
           `CV [%]` = cv,
           `Predicted conc.` = pred,
           `Prediction error [%]` = pred_error,
           `Part of linear regression` = linear) %>%
    mutate(`Prediction error [%]` = 100*`Prediction error [%]`) %>%
    mutate(`Conc.` = signif(`Conc.`, 2))
```

```
  # Store the standard curve plots in a list
  curve_plots[[i]] <-
    ggplot(temp, aes(x = log2(conc), y = log2(mean_ratio))) +
    geom_errorbar(aes(ymax = log2(mean_ratio+std_dev), ymin = log2(mean_ratio-std_dev)), width = 0.2)+
    geom_point(aes(col = linear)) +
    scale_color_manual(values = c("TRUE" = "#2178BF", "FALSE" = "#EB455A")) +
    geom_abline(intercept = -lm_temp$coefficients[1]/lm_temp$coefficients[2],
                slope = 1/lm_temp$coefficients[2]) +
    ggtitle(paste0(peptides$prot[i], ": ", peptides$pep[i])) +
    theme_minimal() +
    xlab(bquote("log"[2]*"(conc. ["*.(params$conc_unit)*"])")) +
    ylab(expression("log"[2]*"(Ratio To Standard)")) +
    theme(legend.position = "none")

# Calculate LOD for the peptide  and the endogenous level of the peptide
loq <- temp %>%
  filter(linear == T) %>%
  group_by(prot, pep) %>%
  summarise(loq = min(conc)) %>%
  mutate(endogenous = 2^(-lm_temp$coefficients[1]/lm_temp$coefficients[2]))

lod <- temp
# Evaluate if the rows used for LOD calculations are sequential
lod$row <- 1:nrow(temp)
lod <- lod %>%
  filter(linear == F)
lod$sequential <- (nrow(temp)-nrow(lod)+1):nrow(temp)

# Remove non-sequential rows and calculate LOD
lod <- lod %>%
  mutate(sequential = row ==sequential) %>%
  filter(sequential == T) %>%
  group_by(prot, pep) %>%
  summarise(lod_ratio = mean(mean_ratio)) %>%
  mutate(lod = 2^((log2(lod_ratio)-lm_temp$coefficients[1])/lm_temp$coefficients[2])) %>%
  select(prot, pep, lod)

# Handle exceptions were the preceeding calcualtions give rise to LOD > LOQ
temp_values <- left_join(lod, loq) %>%
  mutate(lod = ifelse(lod > loq, loq, lod)) %>%
  mutate(loq = ifelse(loq == lod, 3*lod, loq)) %>%
  select(Protein = prot,
         Peptide = pep,
         LOD = lod,
         LOQ = loq,
         `Endogenous level` = endogenous)

# Store the results from the LOQ, LOD and the endogenous level calculations in a list
value_list[[i]] <- temp_values
}

# Convert the list containing LOQ, LOD and endogenous levels into a tibble
value_df <- do.call("rbind", value_list)
```

Standard curves

The following section contains tables and visualization of the standard curves for each peptide included in the dataset. The criteria for a data point to be included in the linear regression determining the linear range are that the data point has a coefficient of variation (CV) below 25% and a bias (deviates from the linear prediction) less than 25%.

The limit of quantifcation (LOQ) is defined as the data point with the lowest concentration included in the linear regression and the limit of detection is calculated as `mean(Ratio To Standard)` for the data points below LOQ. Additionally the endogenous level for each peptide is calculated as the point at which a `Ratio To Standard` of 1 is reached.

In particular cases were the LOQ has been determined as lower than the LOD, using the `mean(Ratio To Standard)` procedure for determining LOD, the value used for LOQ will insted be used as LOD and LOQ will be defined as `3*LOD`.

All concentrations presented in this report are presented with the unit **nM**.

Table 1: Standard curve for EAQLLVIENEVCNHYK from HPRR5000605

Conc.	Ratio To Standard	Standard deviation	CV [%]	Predicted conc.	Prediction error [%]	Part of linear regression
1000.00	0.0169	3e-04	1.78	NA	NA	FALSE
500.00	0.0485	0.00151	3.11	624	24.9	TRUE
250.00	0.15	0.00403	2.69	225	10	TRUE
120.00	0.335	0.00603	1.8	109	13	TRUE
62.00	0.692	0.0188	2.72	56.4	9.79	TRUE
31.00	1.45	0.104	7.16	28.8	7.76	TRUE
16.00	2.73	0.308	11.3	16.3	4.39	TRUE
7.80	5.13	0.905	17.6	9.2	17.8	TRUE
3.90	7.41	1.58	21.3	NA	NA	FALSE
2.00	10.5	1.95	18.5	NA	NA	FALSE
0.98	14.4	5.77	40	NA	NA	FALSE
0.49	19.2	1.89	9.87	NA	NA	FALSE

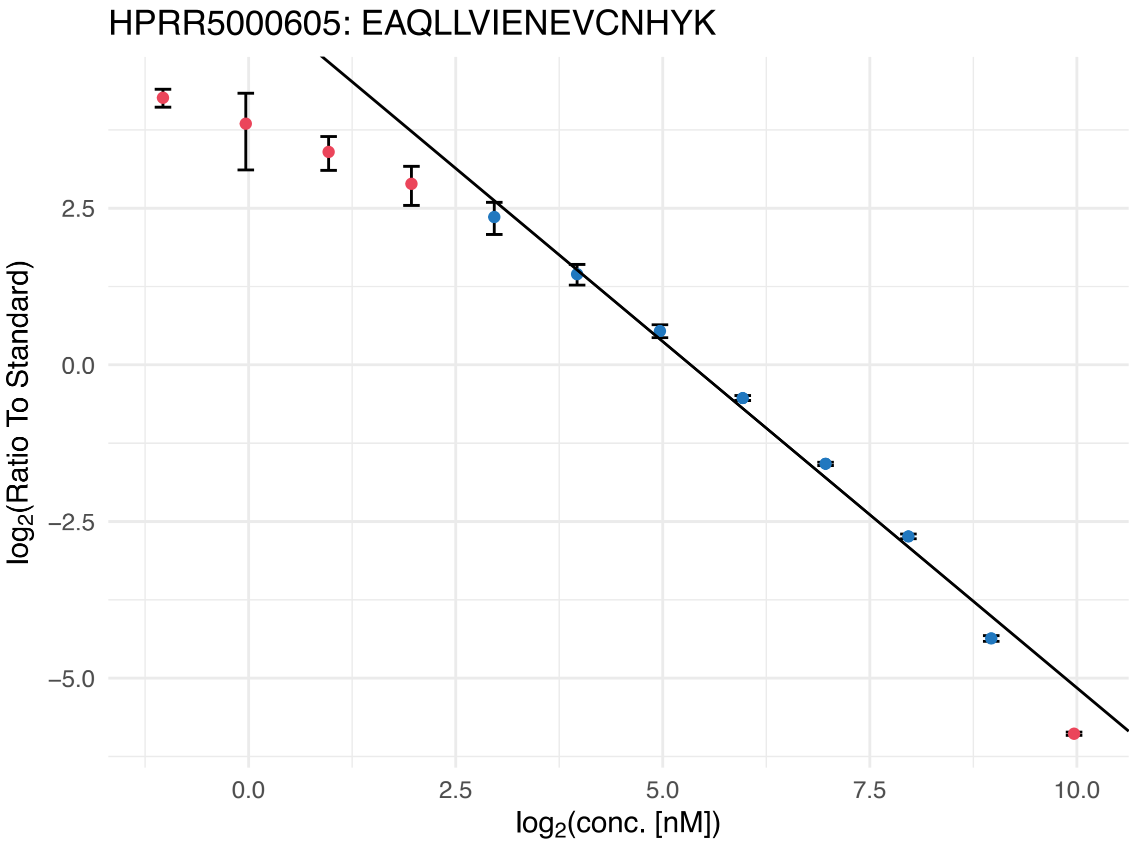

Figure 1: Standard curve for EAQLLVIENEVCNHYK from HPRR5000605

Results for EAQLLVIENEVCNHYK from HPRR5000605

LOD for EAQLLVIENEVCNHYK from HPRR5000605 is **3.5 nM**
LOQ for EAQLLVIENEVCNHYK from HPRR5000605 is **7.8 nM**
The **endogenous level** of EAQLLVIENEVCNHYK from HPRR5000605 is **60 nM**
