# Supplementary figures and images for: Targeted proteomics using stable isotope labeled protein fragments enables precise and robust determination of total apolipoprotein(a) in human plasma
Source: PLoS One. 2023 Feb 15;18(2):e0281772. doi: 10.1371/journal.pone.0281772 (PMC9931122; doi:10.1371/journal.pone.0281772)

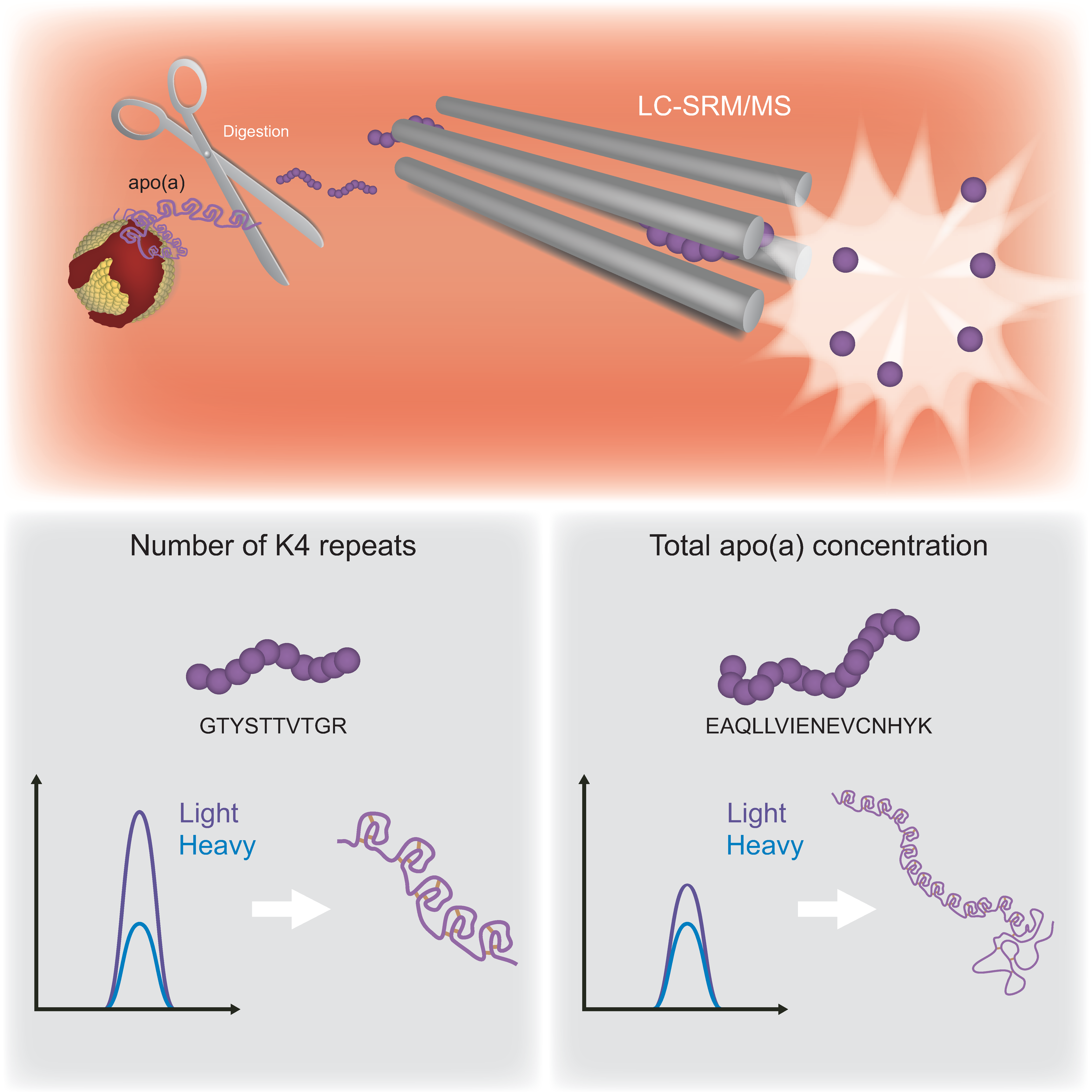

Supplement: S1 Graphical abstract — (TIF) [file pone.0281772.s007.tif]
