# Supplementary material for: Targeted proteomics using stable isotope labeled protein fragments enables precise and robust determination of total apolipoprotein(a) in human plasma
Source: PLoS One. 2023 Feb 15;18(2):e0281772. doi: 10.1371/journal.pone.0281772 (PMC9931122; doi:10.1371/journal.pone.0281772)
Supplement: Attachment — Submitted filename: Response to reviewers.docx [file pone.0281772.s008.docx]

Response to reviewers. Point-to-point in blue

Reviewer #1: Summary:

The authors present a novel approach to quantify apolipoprotein(a) by LC-SRM/MS as a surrogate measure of Lp(a) in plasma using SIS-PREST standards spiked in the samples. The method involves spiking of the samples with SIS-PREST standards, digestion of the proteins, clean-up, and concentration by SPE and injection on LC-MS/MS using a targeted method to measure peptides from 1) the repeatable KIV type 2 domain to determine the average apo(a) isoform size and 2) from the singular Protease Domain to measure the absolute concentration of apo(a). The authors present the development and validation of the method as well as a comparison with an ELISA sandwich assay to measure Lp(a) in a study of 100 individual plasma samples.
The research is timely, and the article is well written, the approach is interesting and perspectives for multiplex analyses are highly relevant to the current context of Lp(a) measurements.

Major comments:

Although the validation of the LC-MS/MS method seems to have been performed thoroughly and according to relevant standards for LC-MS/MS method validation, I think some validation points are still missing from the article in its current state.

In particular, there is no assessment of the LC-MS/MS method accuracy. I assume the authors intended to validate accuracy of their assay using the comparison to an ELISA assay but there are some issues with this approach in my opinion. First, the ELISA kit used for the comparison does not provide results in nmol/L while this is the current recommendation for Lp(a) measurements in clinical routine as stated in the introduction by the authors. Moreover, it is unclear if this assay is traceable to the current reference for Lp(a) and if its accuracy has been validated. Secondly, because the authors used an ELISA comparison assay that does not provide results in nmol/L but in Unit/L, they value assigned these calibrators using their own LC-MS/MS method and the SIS-PREST strategy they developed. I think this is a major issue because they are calibrating both their LC-MS/MS method and the comparison method using the exact same calibration system. As a consequence, the comparison of the results from the two methods only shows how good their calibration system is, not how accurate the LC-MS/MS method is. Therefore, these results are not showing method accuracy. To circumvent this issue, I would recommend using an ELISA assay that provides results in nmol/L and is traceable to the WHO/IFCC SRM-2B reference to perform this comparison again.

A: Thank you for the suggestion. We agree with the reviewer that a standardized independent sample aid with the accurate calibration of the established quantitative system. For the purpose of this study, we therefore specifically selected a commercially available, and calibrated, ELISA to assess the usefulness of the SRM-assay. The Mercodia Lp(a) ELISA 10-1106-01 assay, which we believe is a good benchmark for this purpose, has been benchmarked against three different lots of Bio-Rad Liquicheck Lp(a) control. This serum aliquot is used for quality control and equivalence determination for clinical assays, and consists of a human serum containing stabilized HDL-C, LDL-C, CHOL, TRIG, and apolipoproteins levels of human origin. The Liquicheck resource from BioRad is also used as an assay quality control material to monitor the ongoing precision of clinical laboratory systems (according to FDA). Its Lp(a) content has been determined by five different methodologies to come up with the conversion factor from U/L to mg/dL. As stated by the SRM 2B, stating Lp(a) values in mass units are not applicable due to the size heterogeneity of apo(a). We don’t use the mg/dL, but focus on the linearity between the two assays as pointed out in the manuscript. We have highlighted this limitation in the updated version of the manuscript.

However, benchmarking assays in sera that have been developed in plasma will affect the quantitative accuracy of the assay, and since SRM 2B consists of a pool of sera, this will most likely affect the accuracy negatively and we do not see the added value of using this for determining the accuracy.

I would also recommend using the Deming regression model with associated error to present the comparison data rather than simple plot as in current article.

A: Thank you for the suggestion. We have adopted the Deming regression model for all figures where relevant.

Finally, the LC-MS/MS method includes a SPE step that is known to have potential recovery issues and the authors did not present data on the assessment of digestion completeness and parallelism to validate the method. I think spiking experiments to validate recovery across the whole LC-MS/MS digestion procedure are needed to finalize the validation in addition to the comparison with the ELISA assay in order to assess the LC-MS/MS method accuracy.

A; We agree that this is relevant when spiking standards, and worth addressing when using standards added after the digestion. The repeated 5-day digestion account for biases introduced, and we don’t see any feasible way of studying the separate effects since we use recombinant protein standards and not peptides. The pre- and post-spiking of peptides will not address the potential issue of recovery, and the 5-by-5 experiment presents a summary of all negative effects at once. This was therefore not studied in this experiment but has been studied previously by Oeckl *et al*. 2018). Here, both SIL-proteins, AQUA peptides and SIS-PrESTs were used. The study shows that SIS-PrESTs can account for digestion biases and other pre-analytical steps, and behaves similarly to SIL proteins.

In addition, I think the authors need to clarify and homogenize their statement on the measurement of apo(a) isoform size by LC-MS/MS because it is inconsistent across the article. Indeed, it is of major importance to highlight that measuring a peptide on the KIV2 domain of Lp(a) by LC-MS/MS only provides a measure of the average apo(a) isoform size because 80% of individuals are heterozygous for the LPA gene. It is therefore relevant for only the estimated 20% homozygote individuals.

A; The goal of this assay was to determine the absolute number of KIV2 domains in circulation. We understand that the relative amount of apo(a) would provide even more valuable information, but phasing the total amount into separate heterozygote-specific concentrations is to our knowledge not available by any assay. The ability to determine the concentration of total, or average, number of KIV2 domains, is still of high importance and is a useful tool for understanding the effect of varying KIV2 numbers in a population. We, therefore, believe that this is relevant information for the 80% of individuals that are heterozygous even without the information about their inherited haplotype.

The authors report a cluster of values that behave differently, I think it could be interesting to investigate whether these individuals are homozygote or heterozygote by measuring the apo(a) isoform size using gel electrophoresis as originally reported by Marcovina and colleagues. Moreover, the relevance of the average apo(a) isoform size parameter is still quite debated.

A: We agree that a study addressing the negative correlation observed in multiple studies would be of interest. It has been done in previous studies, now added to the manuscript (PMC3427714), and the tools presented in this article may be used for this type of analysis in a larger cohort. Running gel electrophoresis on this set of samples is not possible and should be done in an orthogonal cohort, for example, while assessing VTE risk.

Here, the authors present a study on 100 individual plasma samples and investigate the correlation between apo(a) isoform size (which should be labelled average isoform size to clarify) and apo(a) concentration.

A: We have changed the terminology to “average” in the instances where we refer to it as the isoform size.

I think this study is interesting but discussion on the results obtained does not mention the issue of the average isoform size and only identifies the small sample size as a limitation to results interpretation. It also only briefly discusses results obtained by other studies who already investigated this comparison. I think the article would greatly benefit from a more thorough discussion on the aforementioned point.

A: We have expanded this section to highlight the limitations of this study.

Finally, I think that overall, the discussion is missing substance and would greatly benefit from some additional points. There are now several methods published on the measurement of Lp(a) by LC-MS/MS and the authors did not discuss the benefits or limitations of their method compared to the others already published.

A: We have made changes to highlight the benefit of using a single assay, that can be combined with other measurements of other apolipoproteins when performed in a multiplex.

The choice of the peptide is also singular compared to the other methods and a more detailed discussion on the choice of this peptide rather than the LFLE peptide most method used would be beneficial. The benefits of the SIS-PREST approach compared to classic SIL peptides could be of interest too.

A: The reason for the choice of peptide has been added to the man uscript. We have also added references to previous studies addressing the benefits of using spiked recombinant standards in comparison to full-length recombinant proteins or peptides.

Minor comments:

1. Some more explanations as to the way SIS-PREST are digested and used would be beneficial in the introduction.

A: We have expanded this section.

2. The way the SIS-PREST calibration standards are prepared is unclear. How many calibration points are there? Are the standards digested in duplicate or triplicate?

A: We have specified this.

3. How was the SIS-PREST standard concentration determined? I think it would be beneficial to detail this information more

A: They are quantified by LC-MS/MS against an amino acid analyzed standard in triplicate measurements. Every standard is quantified by the same method. This has been done for previous publications, and we have done no further work in this context for this article.

4. For the intermediate precision assessment, it seems like there is a drift in the values after 5 days. Did the authors perform tests to assess if the slope is significant? Were the calibration standards freshly prepared for all assays of the precision study? It looks like the standards used for all five assays were the same and that there may be a stability issue.

A: All samples were stored as aliquots for the repeatability test and all reagents have been prepared separately. This outlier (point 5) is more likely from a pipetting error since the accuracy is compromised, rather than the precision that is the 2^nd^ highest of that day. Stability issues would manifest themselves in the freeze-thawing experiment. Because there is no tendency between days 1-4, a variation on this scale between two days is improbable because the standards are stored for months and years.

5. I am quite unclear as to why there are 2 different LC-MS/MS injection methods reported for the reference samples and healthy samples. I think the distinction between these samples and their role in the study is missing in the article.

A: Both equipment configurations were used for the assay development as specified. Due to ethical restrictions and the transfer of material, the healthy cohort that was available was examined in a different lab. This demonstrates again further how adaptable the conventional approach is.

6. Figure 1.A) legend mentions 3 different SIS-PREST peptides but only two are showing on the figure. Figure 1.B) Please also clarify “average” number of K4 repeats.

A: We have specified the average number of K4 repeats in the figure.

7. Figure 2: I think including the 2SD interval on the figures would be beneficial.

A: We have added error bars to this figure.

8. Figure 3: I think showing the identity line and using a Deming regression model would be better to show the comparison data. Also, it is not explicitly explained why the concentrations were log transformed, please clarify.

A: A log-transformed plot is more informative. It creates an evenly weighted distribution that is reasonably symmetrical and crosses the standard curve.

9. Figure 4B and 4C are not very clear and would benefit from some improvements on readability.

We have improved the Figure legend in the new version of the manuscript.

10. Table 2: How was de prediction error determined?

A: This has been added to the manuscript.

11. Graphical abstract: mention “average” number of K4 repeats

A: This is the correct nomenclature as discussed above in regards to average and total.

12. Supplementary Data 1: What is the part on reproducibility about? Was this assessed?

A: All data can be downloaded from Panorama Public, including the reproducibility data in a table format.

13. Supplementary Table: Could these be all merged into a single file for simplicity and more practicality?

A: We have merged all supplementary tables into a single file

Reviewer #2: This manuscript by Hober et al. focusses on the use of mass spectrometry to measure Lp(a) concentrations and apo(a) size polymorphism in human plasma samples. The work is conducted adequately BUT identical studies using the same methodologies have been developed and published in recent years. These studies are not even mentioned in the manuscript except for that of Lassman (2014). The only noticeable difference between this new study and the published ones is that the authors used a distinct proteotypic peptide for apo(a) concentration determination.

Lassman (2014) is cited but only to mention that counting the KIV domains by MS is do-able. It is indeed do-able since it has been done in the past and has been used by many groups for many different studies.

Please refer for instance to the most recent reports (among others):

PMID33517366 (Marcovina 2021)
PMID34314498 (Blanchard 2021)
PMID 32404332 (Blanchard 2020)

A: Thank you for providing additional relevant articles published in the field. We have added them to the bibliography where relevant. We don’t agree with identical studies and have therefore further highlighted the usage of the recombinant protein standards to address biases introduced throughout the workflow.

Reviewer #3: This is a well-written manuscript from a strong and experienced team of which some co-authors I know by relevant publications in proteomic field and HUPO consortium. The authors offered an unconventional approach for the absolute quantification of plasma circulating supramolecular complex, which is LDL-like particle, or Lp(a). The proposed approach permits simultaneous measurement of concentration Apolipoprotein A and estimation of constituent KIV2 repeats, the number of which may vary broadly between subjects. The established method has been validated by traditional ELISA and demonstrated satisfied correlation and overlap within relevant linear range of concentrations.

1. The number of calibrating levels for the developed LC-SRM (page 6, Material and Methods, section “Standard curve”) is not indicated. It is essential to indicate the total number of calibrating levels and the whole range expected to cover by calibration, albeit readers can find this information in Supplementary data. Such information should be defined in the main text first in order to understand calibration range.

A: This has been specified in the manuscript.

2. Why retention time window is so wide (5 minutes) for the developed LC-SRM (Supplementary Table 4)? The wider retention time window, the more concurrent transitions are collected within certain window and less dwell time you obtain per a transition. For example, there are transitions with only 0.866 ms dwell time. Is it enough for the efficient signal accumulation?

A: We have evaluated it based on these parameters and based on our readings, 0.866 ms is enough for efficient single accumulation with satisfactory peak shapes and enough points across peaks. This is the shortest stay time and does not take into account the average time accumulating transitions. Panorama Public provides access to all data per transaction. The standard curves address the measurement's robustness.

The authors used quite robust and reproducible UPLC system giving, probably, sharp peaks with 6-10 s full width (I assume) so they could define a narrow window within 1 minute or less.

A: Yes, a 5-minute window is a large window size, but the low number of transitions tolerate this window size without affecting the sensitivity negatively. The RT drift was less than 30 seconds over a couple of days. This information can be used in a follow up study, when combining the apo(a) peptides with other interesting clinical targets.

3. Quality control of the obtained signal included curation of the “peak shape” as defined in the “Data analysis” section (page 10). It is absolutely unclear and insufficient information of what does the ‘peak shape’ mean? Chromatographic peak consist of many different but well-defined features and the ‘peak shape’ comprises of peak width, asymmetry, tailing, etc. I suggest authors have to provide some more details about the ‘peak shape’ criteria they used for the quality control.

A: We have used rdotp to eliminate peptides decreasing the correlation factor (dopt) between the co-eluting internal standard and endogenous analyte. This has been specified in the manuscript.

4. Obviously, authors employed the Skyline tool for the selection of proper peptides and transitions. In this respect, authors have to indicate such criteria of selection (min-max length, charge states selected (because they used peptide-quantifier with the charge z = 3+), fragment ions type (b, y, a (included?)) and m/z range, definition and monoisotopic or average masses (because they utilized low-resolution instrument), etc. It is essential to apply such criteria in proper way.

A: We have added both the peptide and transition characteristics to the manuscript. This detailed and highly relevant information can also be found on Panorama Public.

5. No doubts as to perfectly done research and development, I suggest the lack of some important information. The authors used two different PrEST as illustrated in Figure 1 (page 11) and stated in the “Results” section (targeted by using two separate SIS recombinant proteins…) which is caused by the interest in specific calculation of K4 domain copy numbers per a molecule and by estimation of the total Apolipoprotein A concentration. However, I found only one of the PrEST constructions in the Supplementary Table 1, which corresponds to 1870-2006 region of canonical Apo A sequence and embodies peptide EAQLL… for particular protein quantification. But where is the second PrEST construction with the embedded K4 domain-specific sequence (GTYS…) for calculation of KIV2 copies?

A: We have revised the Suppl.Table 1 to provide this information and have added the sequence to the list.

6. I accept the defined LOD and LOQ calculated by authors and proposed approach for their calculation. I do not usher to refine and revise them, but I think LOD and LOQ are incorrect and inaccurately calculated. Based on my own and our laboratory experience, we regularly apply recommendations of IUPAC and approved guideline for Evaluation of Detection Capability for Clinical Laboratory Measurement. According such recommendation, you have to establish LOB (limit of blank) first which permits you establishing of LLOD (lowest limit of detection), then LOD and, finally, LOQ. Moreover, you need ‘k’ and ‘b’ coefficient of your linear regression to calculate LOQ correctly: LOQ=LOD+10σc(|b|⁄k)/ Eventually, double LOQ gives Method Limit (ML) value.

A: We will consider this in future publications. Thank you for this suggestion.

7. The selected peptide EAQLLVIENEVCNHYK bears N-terminal glutamic acid which intends to cyclization favorable at already pH=4; internal glutamine and two asparagine residues, which are readily deamidated even at mild conditions; and internal cysteine residue which is modified during alkylation and assumes complete reaction (alkylation) recovery else fraught with inaccurate quantification. It seems that the peptide is hard to control. Why did the authors choose such ‘uncomfortable’ peptide as the main source for quantification? Why, for example, peptide VILGAHQEVNLESHVQEIEVSR was ignored?

A: This is a long peptide (22aa), most likely quadruple charged and has therefore not been considered in our assay development which focuses on +2/3 ions. This becomes more apparent after we have updated the selection criteria, as pointed out by the same reviewer in point 4.

8. The lowest and the highest measured concentrations in assay of ELISA vice the developed LC-SRM seem like outliers (Table 3). If these two points (samples S17 and S20) are eliminated, the corrected R would be more than 0.99. Is it because these measurements are close to limits of detection (upper and lowest) and linearity of LC-SRM and because lower robustness of ELISA (for example, I know that immunoturbidimetry used for Apo A assay is of poor robustness and low accuracy)? Does it mean that the developed LC-SRM is less applicable in clinics compare to ELISA? Does such broad difference (more than 50% in some cases) can be managed in corrective manner to obtain serial results matching stronger between two methods (immunoassay and SRM)?

A: Thank you for suggesting this. We omitted one point from our model and computed a new regression line, and we noted this in the main text.

9. A little more information is essential for samples collected and used for method development, validation and random measurements because Apo A concentration is highly sensitive to fasting before samples collection, smoking before collection, BMI, age and (optional) comorbidities. I do nor require clinical records and history of subjects under consideration, but at least minimal information (as has been touched above) is obligatory to understand possible reason of enormous difference between samples and method utilized (since ELISA is obviously sensitive to deep freezing and thawing because proteins undergo increased denaturation and loss their three-dimensional stability during freezing at low temperatures, which is critically important and may significantly affect the ELISA results. That is why we can occasionally inspect enormous difference between two methods for some instances).

A: We have cited the collection of all samples in the materials and methods and described each analytical step leading up to the analysis accordingly.

Overall, I liked this paper while reading. It was interesting despite the research seems simple. Results of KIV2 copies calculation are intriguing and discovered lack of correlation with the total concentration of Apo A is fascinating. My last question is only of my interest and does not require and modifications in the main text of the paper: why authors went through the synthesis of recombinant proteins fragment (PrEST) instead of preliminary selection proper peptides and their synthesis with terminal isotope-labeled residues? What is the main advantage of PrEST compared to conventional bioinformatic selection and routine synthesis, which are cheaper and easier to follow?

A: Thank you for your advice. In the latest updated version of the manuscript, we have made revisions to emphasize the advantages of using SIS-proteins over SIS-peptides. We have added more references to prior work with SIS-PrESTs to show the technology's potential.
