# Supplementary material for: Targeted proteomics using stable isotope labeled protein fragments enables precise and robust determination of total apolipoprotein(a) in human plasma
Source: PLoS One. 2023 Feb 15;18(2):e0281772. doi: 10.1371/journal.pone.0281772 (PMC9931122; doi:10.1371/journal.pone.0281772)
Supplement: Attachment — Submitted filename: Review_2.1.docx [file pone.0281772.s009.docx]

The authors satisfied all questions addressed. I have also reviewed data submitted to Panorama (as declared in the ‘Data Availability” statement) and I was satisfied by the presented results concerning signal stability and robustness. In the revised paper the Authors also unfurled the advantage of SIS PrEST approach as I asked them before, and added relevant references. I hope, the Authors would update the review about this technique and achievements in someone future specific paper.

The Authors also made essential amendments in the Supplementary to display accurately the design of SIS PrEST experiment as I claimed before. I also marked more details regarding the comparison of efficiency between ELISA and SRM approaches and comprehensive calculation of the true concentration of calibrants. I have no doubts and specific question regarding the revised version of manuscript. Only minor corrections and amendments are needed. Specifically:

1. ‘SI-units’ abbreviation appeared the first time in page 13 and should be defined;
2. ‘…protein-level spike-ins of standards rather…’: spike-ins’ is probably mistyped and should be ‘spike-in’;
3. ‘Regionala etikprövnignsnämnden’ on page 6: the ‘Etikprövnignsnämnden’ should begin from the capital letter “E” as on page 5;
4. I would suggest to place a part the ‘Results’ section (Peptide ions with a length of 5-25 amino acids and a precursor charge state z = 2,3 and product ion charge state z = 1,2 were screened. Only b- and y-ions were included in the analysis. (33, 34)) in the ‘Material and Methods’ section as a better place.
5. ‘Discussion’ section, page 17: ‘…variance in KIV2 repeats, it was not possible to detect the previously observed negative correlation between number of KIV2 repeats and apo(a) concentrations previously observed (10).’ – I think, one of ‘previously observed’ is redundant.
