# Supplementary material for: Targeted proteomics using stable isotope labeled protein fragments enables precise and robust determination of total apolipoprotein(a) in human plasma
Source: PLoS One. 2023 Feb 15;18(2):e0281772. doi: 10.1371/journal.pone.0281772 (PMC9931122; doi:10.1371/journal.pone.0281772)
Supplement: Attachment — Submitted filename: Response to reviewers.docx [file pone.0281772.s010.docx]

# Response to reviewers. Point-to-point in blue

Review Comments to the Author

Please use the space provided to explain your answers to the questions above. You may also include additional comments for the author, including concerns about dual publication, research ethics, or publication ethics. (Please upload your review as an attachment if it exceeds 20,000 characters)

Reviewer #1: Although the authors addressed some of my previous comments and suggestions, I do not think the quality of the article has sufficiently improved yet to be released for publication. Some of my main concerns have not been addressed and I think the current article is still missing some important discussion points:

1. I understand the authors response to my comment on the choice of the comparison ELISA assay for the validation of their LC-MS/MS method although I do not agree. Assessing accuracy means assessing closeness of agreement of the value measured with the new method versus the true value of the analyte in the sample. The true value cannot be measured and only estimated within an error range. Metrology institutes and international instances for method accuracy work worldwide on developing standards, measured with highly precise and highly accurate methods, traceable to the international system of units to determine a value as close as possible to the true value for an analyte so that secondary methods like ELISA can use them as calibrators and be ensured accuracy. The SRM-2B is a very imperfect matrix-based material, a pool of plasma indeed, but it has one advantage: it has an assigned reference value, internationally agreed on and used as a general anchor for most Lp(a) assays worldwide. Using it as a comparison point, although it will not be accuracy in its strictest sense since SRM-2B is not traceable to the SI, is still the best comparison point available for Lp(a).

My suggestion and comments were not this though. SRM-2B is a precious material not easily accessible for studies and could understandably not be used for this research. However, multiple commercial assays for ELISA are directly traceable to SRM-2B, and provide results directly in nmol/L, which is the best solution. The fact that the authors used a benchmark assay for their comparison is not my point. I fully trust the good sense of the researchers to pick an assay performing well. My point was that they should chose an assay traceable to the SRM-2B, the only available international reference for Lp(a), so that they compare their LC-MS/MS method to an ELISA which accuracy has been proven and certified. Comparing to an ELISA without stating its traceability does not provide any information on accuracy, it only provides information that the LC-MS/MS is comparable to this specific ELISA.

Based on the authors response, I seem to understand that the Mercodia ELISA assay they used was verified in some ways against commercial controls. The traceability of this ELISA kit to the SRM-2B, if available, should be mentioned clearly in the main text. If the ELISA used is not traceable to the SRM-2B, this issue should be thoroughly discussed in the main text and the associated limitation regarding claims for accuracy clearly stated. Similarly, the controls used to check this test and their traceability should be mentioned in the main text.

I strongly insist on addressing this concern within the main text. Authors mention they updated this limitation in the main text, I do not see these changes. I think they should mention their benchmark so that they can defend within the main text and to all readers the choice of this ELISA and the reason why they are confident in their assessment of accuracy by comparing their LC-MS/MS to this ELISA specifically.

A: Thank you for the for the comment. The traceability of the assay that was previously included in response to reviewers has been added to the main text to highlight the validity of the chosen ELISA for the comparison experiment.

2. I disagree with the authors’ response regarding the measure of average apo(a) isoform size. I did understand the goal of measuring the total number of KIV-2 circulating in blood and measuring this is, indeed, interesting in some ways. However, what is important about apo(a) isoform size is that it will impact Lp(a) particle size, which will have consequences on its metabolism. What is relevant is therefore the size of the particles circulating, of which apo(a) isoform is only a surrogate measure. Knowing the total average amount of KIV-2 does not provide this information.

The reason I insist is precisely that people tend to get confused on this point, which I think us as researchers are responsible for making clear. I do not question the quality of the method to measure the average apo(a) isoform size but ask that this nuance in terms of clinical usefulness be mentioned in the main text clearly.

Moreover, there do exists a method to assess the distribution of apo(a) isoform size within a sample: gel electrophoresis as mentioned previously by the authors. I was not requesting that the authors run this method on their sample, and I understand it is not feasible for these study samples given small amounts available. However, mentioning the limitation of average apo(a) isoform size vs actual apo(a) isoform size distribution measurement and stating these limitations in the discussion is feasible. There is ample available bibliography on this subject.

A: This has been expanded upon in the revised version of the manuscript.

3. The authors state in their response that they expanded the discussion to highlight the limitations of this study. I do not see the difference in the revised manuscript. I maintain my comment that the discussion in its current form still lacks substance.

First of all, as mentioned by reviewer 2, the authors do not cite nor discuss work previously published on LC-MS/MS methods to measure apo(a). The mention of previously published methods only appears in the introduction and is quite short and feels very expeditive. I think expending on what the 3 other groups with existing methods have already done, at least in a few sentences, would be a good idea for the introduction. Even though this method is definitely interesting with SIS-PrEST technology and its value-added for quantification, the way the introduction is written sounds like this is the first ever LC-MS/MS method on Lp(a), which it is not.

Overall, my point is: ok the SIS-PrEST approach is an interesting approach, but what is new for Lp(a)? In the end, what is the point of using this method versus another multiplex one developed by Blanchard and colleagues for example who use cheap and easy access SIS peptides, have good accuracy and quick method running in clinical setting already? Why go through the hurdle of using SIS-PrEST? How expensive is the use of SIS-PREST? You mention its potential use in clinical setting, would that be financially robust and feasible? What are the performances of this specific method compared to that of Marcovina and colleagues in terms of reproducibility, turn-around time, precision? What about discussion on the choice of this long peptide, potentially unstable as very interestingly pointed out by reviewer 3? What about digestion completeness and accuracy bias due to different steps? The authors answered to me that SIS-PrEST takes this into account, I think this answer should typically be included in the discussion as it is relevant to the readers too. What about the limitations of this method in terms of practicality in clinical laboratories? I mentioned prices of the SIS-PrEST, what about ease of access? Batch to batch reproducibility and purity? Stability over long period of storage?

In my opinion, all these points should be extensively mentioned and discussed in the article to improve its quality, interest, and general impact to the scientific community.

A: We have expanded this part of the introduction to highlight the rigorous work done by other groups in this area and to highlight the benefits brought about by using the SIS PrESTs over a standardized SIL peptide-based workflow. Additionally, we have expanded on these aspects in the discussion to a larger extent, rather than citing previous work.

Minor comments:

1. Page 14 – Figure 3: Where is the discussion regarding the fact that the error range is quite large and that there is significant deviation from unity at low concentrations?

A: This has been addressed in the updated version.

2. Page 16 – Line 2 (discussion): Average isoform size.

A: This has been addressed in the updated version.

Reviewer #2: The authors have satisfactorily answered to my initial comments. I than them for their response and hope that their article will meet its readership.

Reviewer #3: The authors satisfied all questions addressed. I have also reviewed data submitted to Panorama (as declared in the ‘Data Availability” statement) and I was satisfied by the presented results concerning signal stability and robustness. In the revised paper the Authors also unfurled the advantage of SIS PrEST approach as I asked them before, and added relevant references. I hope, the Authors would update the review about this technique and achievements in someone future specific paper.

The Authors also made essential amendments in the Supplementary to display accurately the design of SIS PrEST experiment as I claimed before. I also marked more details regarding the comparison of efficiency between ELISA and SRM approaches and comprehensive calculation of the true concentration of calibrants. I have no doubts and specific question regarding the revised version of manuscript. Only minor corrections and amendments are needed. Specifically:

1. ‘SI-units’ abbreviation appeared the first time in page 13 and should be defined;

A: We have rephrased this sentence to avoid confusion.

2. ‘…protein-level spike-ins of standards rather…’: spike-ins’ is probably mistyped and should be ‘spike-in’;

A: This has been addressed in the updated version.

3. ‘Regionala etikprövnignsnämnden’ on page 6: the ‘Etikprövnignsnämnden’ should begin from the capital letter “E” as on page 5;

A: This has been addressed in the updated version, and both instances have been revised to be spelled with lowercase “e”.

4. I would suggest to place a part the ‘Results’ section (Peptide ions with a length of 5-25 amino acids and a precursor charge state z = 2,3 and product ion charge state z = 1,2 were screened. Only b- and y-ions were included in the analysis. (33, 34)) in the ‘Material and Methods’ section as a better place.

A: This has been addressed in the updated version.

5. ‘Discussion’ section, page 17: ‘…variance in KIV2 repeats, it was not possible to detect the previously observed negative correlation between number of KIV2 repeats and apo(a) concentrations previously observed (10).’ – I think, one of ‘previously observed’ is redundant.

A: This has been addressed in the updated version.

7. PLOS authors have the option to publish the peer review history of their article ([what does this mean?](https://journals.plos.org/plosone/s/editorial-and-peer-review-process#loc-peer-review-history)). If published, this will include your full peer review and any attached files.


If you choose “no”, your identity will remain anonymous but your review may still be made public.


**Do you want your identity to be public for this peer review?** For information about this choice, including consent withdrawal, please see our [Privacy Policy](https://www.plos.org/privacy-policy).

Reviewer #1: No

Reviewer #2: No

Reviewer #3: **Yes:** Arthur T. Kopylov
